# Laboratory evolution in *Novosphingobium aromaticivorans* enables rapid catabolism of a model lignin-derived aromatic dimer

**DOI:** 10.1128/aem.02081-24

**Published:** 2025-01-23

**Authors:** Marco N. Allemann, Ryo Kato, Dana L. Carper, Leah H. Hochanadel, William G. Alexander, Richard J. Giannone, Naofumi Kamimura, Eiji Masai, Joshua K. Michener

**Affiliations:** 1 Biosciences Division, Oak Ridge National Laboratory 6146 , Oak Ridge, Tennessee, USA; 2 Department of Materials Science and Bioengineering, Nagaoka University of Technology 52756 , Nagaoka, Niigata, Japan; 3 Center for Bioenergy Innovation, Oak Ridge National Laboratory 6146 , Oak Ridge, Tennessee, USA; Shanghai Jiao Tong University, Shanghai, China

**Keywords:** lignin, *Novosphingobium aromaticivorans*, GGE

## Abstract

Lignin contains a variety of interunit linkages, leading to a range of potential decomposition products that can be used as carbon and energy sources by microbes. β-O-4 linkages are the most common in native lignin, and associated catabolic pathways have been well characterized. However, the fate of the mono-aromatic intermediates that result from β-O-4 dimer cleavage has not been fully elucidated. Here, we used experimental evolution to identify mutant strains of *Novosphingobium aromaticivorans* with improved catabolism of a model aromatic dimer containing a β-O-4 linkage, guaiacylglycerol-β-guaiacyl ether (GGE). We identified several parallel causal mutations, including a single nucleotide polymorphism in the promoter of an uncharacterized gene that roughly doubled the growth yield with GGE. We characterized the associated enzyme and demonstrated that it oxidizes an intermediate in GGE catabolism, β-hydroxypropiovanillone, to vanilloyl acetaldehyde. Identification of this enzyme and its key role in GGE catabolism furthers our understanding of catabolic pathways for lignin-derived aromatic compounds.

**IMPORTANCE:**

Lignin degradation is a key step for both carbon cycling in nature and biomass conversion to fuels and chemicals. Bacteria can catabolize lignin-derived aromatic compounds, but the complexity of lignin means that full mineralization requires numerous catabolic pathways and often results in slow growth. Using experimental evolution, we identified an uncharacterized enzyme for the catabolism of a lignin-derived aromatic monomer, β-hydroxypropiovanillone. A single nucleotide polymorphism in the promoter of the associated gene significantly increased bacterial growth with either β-hydroxypropiovanillone or a related lignin-derived aromatic dimer. This work expands the repertoire of known aromatic catabolic genes and demonstrates that slow catabolism of lignin-derived aromatic compounds may be due to misregulation under laboratory conditions rather than inherent catabolic challenges.

## INTRODUCTION

Lignin is the second most abundant natural polymer and one of the three major components of plant cell walls (1). The lignin polymer is derived from three main monomer units, *p*-coumaryl alcohol, coniferyl alcohol, and sinapyl alcohol, which differ based on the presence and number of methoxy units on the aromatic ring. In plants, these monomer building blocks are coupled via radical mechanisms to generate a wide variety of interunit linkages (2). Breakdown of lignin in the environment is thought to be performed mainly by fungi, which excrete extracellular peroxidases and laccases that cleave high molecular weight polymeric lignin into lower molecular weight aromatic compounds (3). These small soluble products can be further mineralized by other microbes. In bacteria, pathways have been described for the conversion of diverse aromatic dimers into their constituent monomers, which can then be funneled into core catabolic pathways such as the various protocatechuate ring cleavage pathways (4, 5). These degradation pathways can also be used to valorize depolymerized lignin into value-added bioproducts (6, 7).

**Fig 1:**
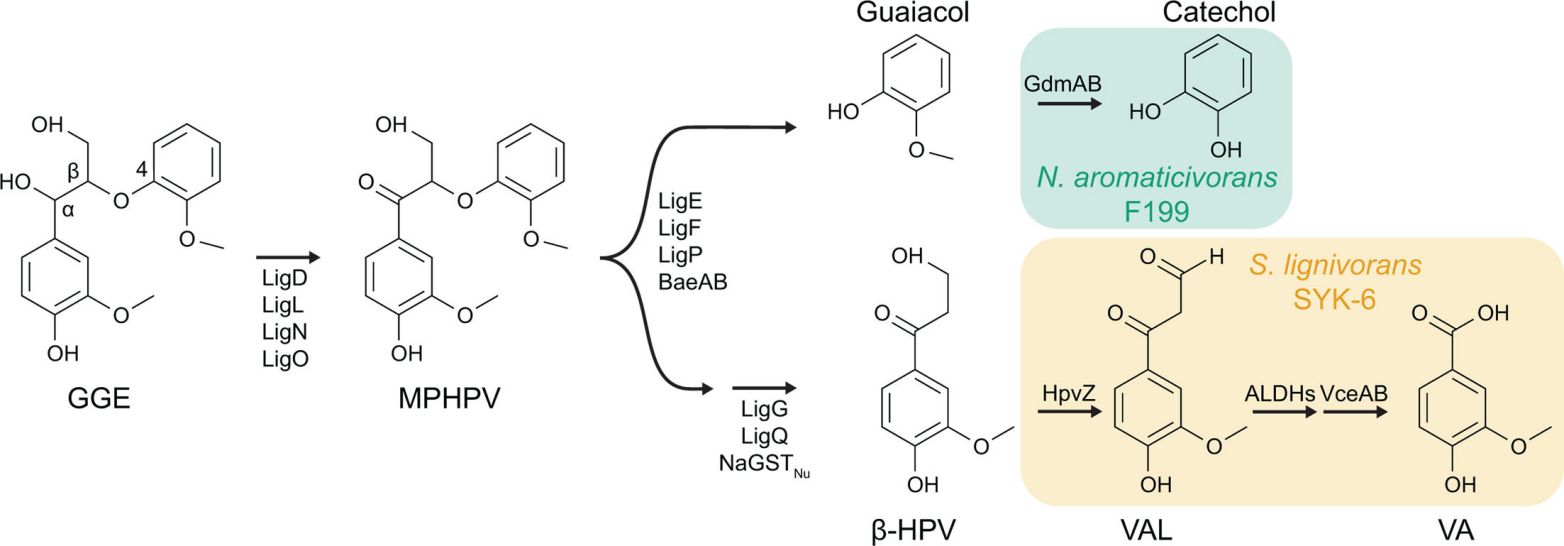
GGE catabolic pathways in two model lignin-degrading bacteria. The alcohol group at the α position of GGE is oxidized to form MPHPV. Various β-etherases cleave the β-ether group yielding β-HPV and guaiacol. Previous works identified catabolic pathways for guaiacol in *N. aromaticivorans* F199 (17) and β-HPV in *S. lignivorans* SYK-6 (14, 20). For simplicity, stereochemistry of GGE and MPHPV is not shown. GGE, guaiacylglycerol-β-guaiacyl ether; MPHPV, α-(2-methoxyphenoxy)-β-hydroxypropiovanillone; β-HPV, β-hydroxypropiovanillone; VAL. vanilloyl acetaldehyde; VA, vanillic acid. Enzymes: LigD, LigL, LigN, and LigO, short-chain dehydrogenase/reductases; LigE, LigF, LigP, BaeAB; β-etherases; LigG, LigQ, NaGST_Nu_ glutathione *S*-transferase; GdmAB, guaiacol *O*-demethylase; HpvZ, β-HPV oxidase; ALDHs, aldehyde dehydrogenases; VceA, VAA-converting enzyme; VceB, vanilloyl-CoA/syringoyl-CoA thioesterase.

To date, pathways for the catabolism of lignin-derived aromatic dimers connected by β-O-4 linkages have been the best characterized (Fig. 1). This linkage typically comprises up to 40%–50% of the inter-unit linkages found in lignin (1). A pathway for the catabolism of dimers with β-O-4 linkages was first discovered over three decades ago in the Alphaproteobacterium *Sphingobium lignivorans* SYK-6 (hereafter “SYK-6”) using the model compound guaiacylglycerol-β-guaiacyl ether (GGE) and has been extensively characterized at the genetic and biochemical level (8–14). More recently, additional enzymes for the degradation of GGE and related intermediates have also been characterized in *Novosphingobium aromaticivorans* F199 (hereafter “F199”) (15–17) as well as other bacteria (18, 19). As shown in Fig. 1, GGE catabolism in both F199 and SYK-6 proceeds via a set of conserved biochemical steps to produce the intermediates β-hydroxypropiovanillone (β-HPV) and guaiacol. In SYK-6, the β-HPV intermediate is funneled into the protocatechuate 4,5-cleavage pathway, and several of the necessary enzymes have recently been described (14, 20), while guaiacol is not assimilated. In contrast, previous work using F199 growing with GGE demonstrated that guaiacol was rapidly consumed but β-HPV accumulated transiently (15).

While both SYK-6 and F199 can grow with GGE as the sole carbon and energy source, neither wild-type strain grows rapidly under these conditions. For example, F199 takes approximately 15 days to reach the stationary phase during growth with 3 mM GGE (15). We previously used experimental evolution to isolate a mutant of F199, which we termed JMN2, that grows more rapidly with GGE, reaching a stationary phase in approximately 3 days (21). Using barcoded transposon insertion sequencing in JMN2, we discovered a Rieske monooxygenase, GdmA, that demethylates guaiacol to catechol (17). Catechol can then be assimilated using a pathway for oxidative 1,2-cleavage of catechol that is natively present in F199. This newly described pathway explains how F199 catabolizes the guaiacol produced from GGE degradation.

Since F199 was shown to accumulate stoichiometric quantities of β-HPV during growth with GGE but then fully catabolize β-HPV (15), we hypothesized that a pathway for β-HPV catabolism was present in this strain but inefficiently regulated under laboratory conditions. However, F199 does not contain a close homolog of the *hpvZ* gene that encodes the first enzyme for β-HPV catabolism in SYK-6 (20), so the identity of this potential catabolic pathway was unknown.

In this work, we describe further laboratory evolution experiments of F199 grown with GGE as the sole carbon and energy source. These evolution experiments yielded strains with a range of growth phenotypes, including one strain with more than a twofold increase in cell density at the stationary phase during growth with GGE compared to JMN2. Resequencing, mapping, and comparison of mutations across these evolved F199 strains led to the discovery of a β-HPV-processing enzyme that we designate as HpvY. Heterologous expression in *Escherichia coli* demonstrated that HpvY converts β-HPV to vanilloyl acetaldehyde (VAL).

## MATERIALS AND METHODS

### Bacterial strains and growth conditions

Stains used in this work are described in Table 1. *Escherichia coli* strains were routinely grown at 37°C in Lysogeny Broth (LB) Miller unless stated otherwise. *Novosphingobium aromaticivorans* F199 strains were grown at 30°C in R2A medium (Teknova) unless noted otherwise. For the growth of *N. aromaticivorans* strains in a minimal medium with defined carbon sources, DSM 457 medium was used as described previously (22). For solid media, agar was included at 15 g/L. The antibiotics kanamycin (50 µg/mL) and streptomycin (100 µg/mL) were used as required. Diaminopimelic acid (DAP) was added to LB as needed (60 µg/mL). All aromatic carbon substrates were dissolved in dimethylsulfoxide and added to DSM457 for 1 g/L (wt/vol) final concentration. Due to the toxicity of guaiacol, it was added at a concentration of 0.25 g/L (2.0 mM).

**TABLE 1:** Strains and plasmids used in this study

Strain or plasmid	Description^*a*^	Source
*Escherichia coli* strain		
WM6026	*lacI*^q^, *rrnB3*, Δ*lacZ4787*, *hsdR514*, Δ*araBAD567*, Δ*rhaBAD568*, *rph-1*, *att*λ::pAE12(Δ*oriR6K-cat*::Frt5), Δ*endA*::Frt, *uidA*(ΔMluI)::*pir*, *attHK*::pJK1006D(*oriR6K-cat*::Frt5; *trfA*::Frt) *dap*	(23)
*Novosphingobium aromaticivorans* strains		
F199	Wild-type strain; DSM12444	DSMZ 12444
JMN2	GGE-evolved F199	(21)
JMN123	GGE-evolved F199	This study
JMN122	GGE-evolved F199	This study
JMN121	GGE-evolved F199	This study
JMN30	JMN2, ∆*ligE* (*RS12100*)	This study
JMN142	JMN123, ∆*ligE* (*RS12100*)	This study
JMN151	JMN123 with an additional 6.6 kb deletion in pNL1	This study
JMN158	JMN123, ∆*catA* (*RS20025*)	This study
JMN159	JMN123, ∆*hpvY* (*RS19130*)	This study
JMN160	JMN123. ∆*catB* (*RS20015*)	This study
JMN161	JMN123, ∆*gdmA* (*RS07455*)	This study
*Sphingobium lignivorans* strain		
SYK-6	Wild-type strain	NBRC 103272
Plasmids		
pAK405	Allele exchange plasmid for sphingomonads, Kan^R^, *rpsL* (Sm^S^)	(24)
pJM328	pAK405, ∆*ligE*	This study
pJM489	pAK405, ∆*hpvY*	This study
pJM301	pAK405, ∆*catA*	(17)
pJM300	pAK405, ∆*catB*	(17)
pJM319	pAK405, ∆*gdmA*	(17)
pET-16b	Expression vector; T7 promoter; Ap^R^	Novagen
p16hpvY	pET-16b with a 1.7-kb NdeI-BamHI PCR-amplified fragment carrying *hpvY (RS19130*)	This study

^*a*^ Kan^R^, Sm^S^, and Ap^R^ indicate resistance to kanamycin, sensitive to streptomycin, and resistance to ampicillin, respectively.

### Laboratory evolution experiment

Triplicate cultures of wild-type *N. aromaticivorans* F199 were inoculated from a single colony into DSM 457 with 2 g/L glucose and grown overnight at 30°C with shaking at 200 RPM. Cells were harvested by centrifugation and washed three times with DSM 457 lacking a carbon source and were inoculated at a 100-fold dilution into DSM 457 medium supplemented with GGE (1 g/L) as a sole carbon source and incubated at 30°C with shaking at 200 RPM. Cultures were assessed weekly and diluted 100-fold into fresh DSM 457 with 1 g/L GGE when growth was visually apparent by eye (~OD_600_ 0.1) and significantly above the non-inoculated control. After approximately 2 months of growth and a total of five transfers, aliquots of the GGE-grown cultures were streaked onto R2A agar for the isolation of single colonies. Several purified colonies from each of the three evolutionary lineages were further screened for their abilities to grow with GGE, and the isolate derived from each lineage with the highest final optical density was chosen for further analysis.

### Resequencing and identification of mutations

Genomic DNA was isolated from the evolved mutants using a Promega Wizard genomic DNA kit following the manufacturer’s guidelines for Gram-negative bacteria with minor modifications; a second centrifugation step was included after the protein precipitation to improve DNA quality. Genomic DNA was prepared for Illumina library construction, and libraries were sequenced on the MiSeq Illumina platform to generate paired-end 300 bp reads using V3 chemistry. Sequence analysis and variant calling were performed in Geneious Prime (version 2021.0.3). Raw reads were trimmed with BBDuk plugin (version 1.0) with default settings. Trimmed reads were mapped to the *N. aromaticivorans* F199 reference genome using the bowtie plugin (version 7.2.1). To identify single-nucleotide polymorphisms, the minimum variant frequency was set to 0.8. Mutations of interest were further verified by PCR and Sanger sequencing of purified products.

### Nanopore sequencing and genome assembly

A total of 10 ng of genomic DNA was amplified using the GenomiPhi v2 whole-genome amplification kit (Cytiva, Marlborough, MD, USA), then the resulting DNA was simultaneously cleaned, concentrated, and size selected to remove fragments under 1 kbp by performing a bead cleanup with 0.4× volumes of HighPrep PCR cleanup beads (MagBio, Gaithersburg, MD, USA). A total of 1.5 µg of this amplified DNA (hereto referred as wga DNA) was digested by 1.5 µL of T7 endonuclease (New England Biolabs, Ipswich, MA, USA) in a 50 µL reaction for 1 h and then the digested wga DNA along with the native, unamplified DNA (hereto referred as nat DNA) was cleaned, concentrated, and size selected against fragments under 5 kbp using HighPrep PCR beads in a custom buffer containing 1.25 M NaCl and 20% PEG-8000 (25). The resulting DNA was used in conjunction with the Oxford Nanopore Technology (ONT, Oxford, UK) Rapid Barcoding Kit SQK-RBK004 to produce a sequencing library with nat and wga DNA barcoded separately, which was then loaded onto a MinION R9.4.1 flow cell driven by a Mk. IIB MinION device. The raw data were basecalled using the ONT Guppy basecaller on an HP Z8 workstation equipped with an NVIDIA Quadro RTX 4000 GPU with 8 GB of memory. The resulting reads for each sample underwent quality control using Filtlong version 0.2.1 (https://github.com/rrwick/Filtlong) to remove fragments under 1 kbp and then to remove the bottom 5% of reads by quality. The resulting nat reads were used with Trycycler version 0.5.1 (26) along with the assemblers Raven version 1.5.3 (27), Miniasm + Minimap version 0.3-r179 (28), and Flye version 2.9 (29). This draft assembly was first polished using Medaka version 1.5 (https://github.com/nanoporetech/medaka/) together with the nat reads and then Medaka polishing was repeated using the wga reads. Short-read polishing was then performed first with Polypolish version 0.4.3 (30) and then with Pilon version 1.24 (31) to remove common errors associated with long-read assemblies (e.g., disagreement in homopolymer length).

### Targeted gene deletion mutagenesis

Chromosomal deletions in *N. aromaticivorans* were constructed using previously described methods with minor alterations (22). In-frame deletions were generated by allelic exchange using vector pAK405 (24). Briefly, homology arms containing 500–700 bp regions upstream and downstream of genes to be deleted were synthesized and cloned into pAK405 (Genscript, Piscataway, NJ, USA). Constructs were mobilized into *N. aromaticivorans* via conjugation using the *E. coli* DAP auxotroph strain WM6026 with selection on R2A medium containing kanamycin. Exconjugants were streaked to single colonies once from selection plates and grown overnight in R2A in the absence of kanamycin selection. Aliquots of the overnight culture were plated on R2A + streptomycin agar for counterselection against the integrated plasmid. Streptomycin-resistant colonies were patched to R2A + kanamycin + streptomycin and R2A streptomycin to screen for kanamycin sensitivity. Kanamycin-sensitive colonies were screened for gene deletions by colony PCR.

### Growth rate measurements

**Fig 2:**
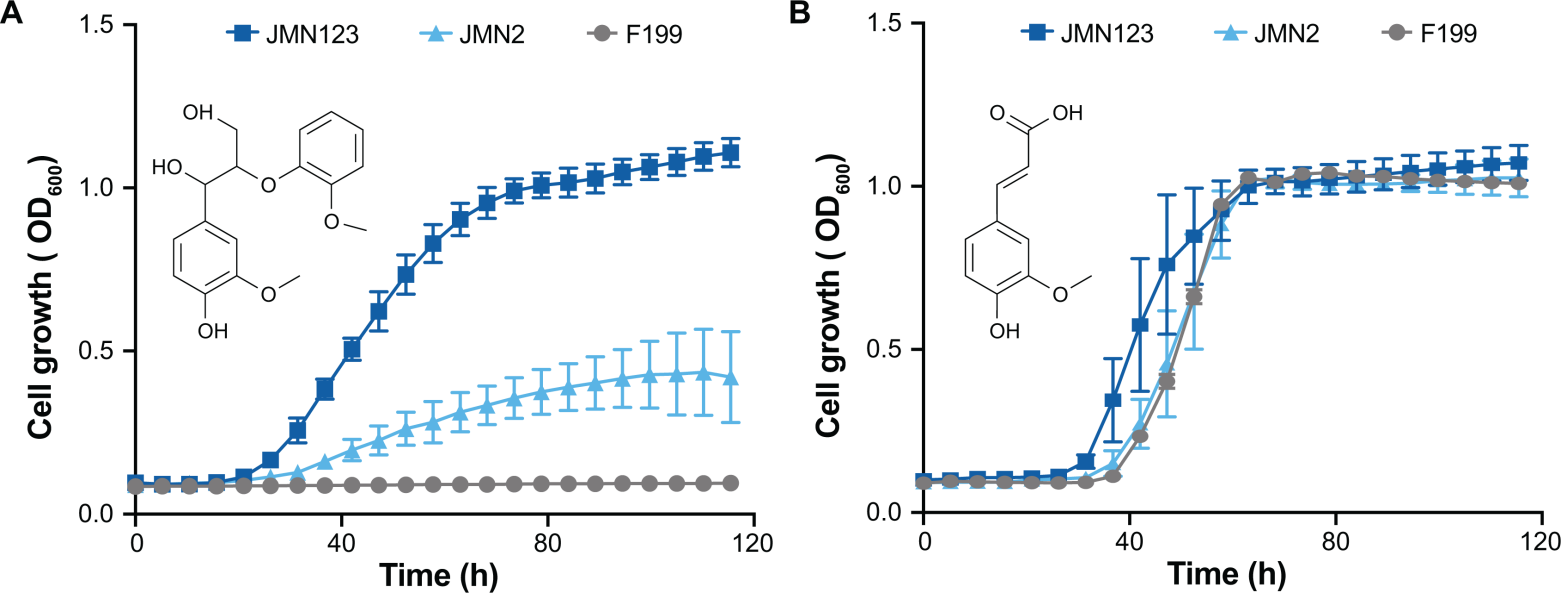
Adaptive laboratory evolution of *N. aromaticivorans* F199 for improved growth on GGE as a sole carbon source. (**A**) Growth of wild-type and evolved strains with 1 g/L GGE (3.1 mM) as the sole carbon and energy source. (**B**) Growth of wild-type and evolved strains with 1 g/L ferulate (5.1 mM) as the sole carbon and energy source. Growth curves shown are the means of at least three independent experiments. Error bars show one standard deviation, calculated from three independent experiments.

For the data shown in Fig. 2 and Fig. S1, S3, S5, and S7, strains were grown to stationary phase overnight in DSM 457 minimal medium with 2 g/L glucose. Cells were pelleted (1 min at 10,000 × *g*) and washed once with DSM 457 lacking a carbon source before being diluted 100-fold into fresh medium containing the appropriate carbon source and grown as triplicate 1 mL cultures in 48-well plates (Greiner Bio-One, Kremsmünster, Austria). The growth was monitored by measuring the optical density at 600 nm (OD_600_) in an Epoch 2 plate reader (Agilent, Santa Clara, CA, USA). The growth rates were calculated using the R package growthcurver (32).

**Fig 3:**
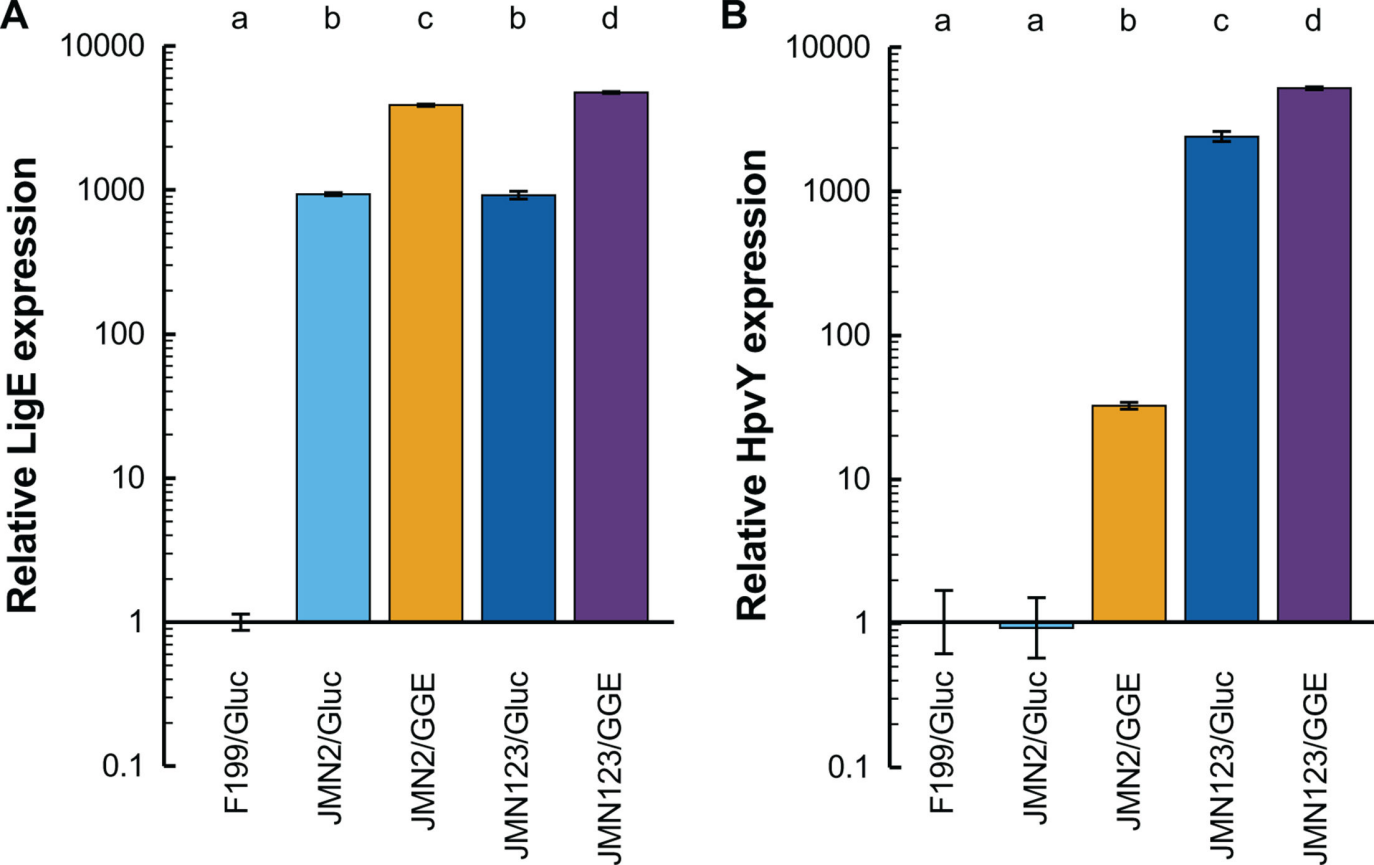
Mutations observed during experimental evolution increase the expression of (**A**) LigE and (**B**) HpvY. The indicated strains were grown in DSM457 with 1 g/L of the indicated carbon source, either glucose or GGE. Error bars show one standard deviation, calculated from three biological replicates. Letters indicate groups with statistically significant differences in enzyme expression (*P* < 0.05).

For the data shown in Fig. 3, strains F199, JMN2, JMN123, JMN159, and SYK-6 were cultured in LB for 24 h at 30°C. The cells were harvested by centrifugation at 14,000 × *g* for 1 min at 4°C, washed twice with 3 mL of Wx medium (33), and then suspended in the same medium. The cell suspensions were inoculated into Wx medium containing 2 mM GGE or HPV to an optical density at 600 nm (OD_600_) of 0.1 and incubated for 100 h at 30°C with linear shaking at 567 cycles per minute using an EPOCH microplate reader (Agilent, Santa Clara, CA, USA). The OD_600_ was measured continuously.

### Metabolite accumulation assays

Strains were grown to stationary phase overnight in DSM 457 medium with 2 g/L glucose. Cells were harvested by centrifugation and washed several times with DSM 457 lacking a carbon source. Cells were then diluted 100-fold into fresh DSM 457 medium containing 1 g/L GGE and incubated at 30°C. At the indicated times, ~1 mL aliquots of the culture were removed, cells were pelleted, and the supernatant was collected and passed through a 0.22 µm polyethersulfone filter. Filtered supernatant samples were then analyzed by LC-MS to monitor intermediate product accumulation across the strains over time. For each sample, 5 µL of supernatant was split-loaded onto an in-house pulled nanospray emitter (75 µm inner diameter) packed with 15 cm of C18 resin (1.7 µm Kinetex; Phenomenex) and separated over a 15 min reversed-phase gradient using a Vanquish HPLC interfaced directly to a Q Exactive Plus mass spectrometer (Thermo Scientific) (21). Eluting analytes were measured by the Q Exactive operating in negative ion mode monitoring a mass-to-charge range of 100–500 *m*/*z*; resolution 35,000; three microscan spectrum averaging. Peak areas were extracted for MPHPV (317.1031 [M-H]) and β-HPV (195.0663 [M-H]) pathway intermediates via Skyline software (34), and the areas were compared across samples to assess strain bottlenecks.

### Preparation of resting cells

Cells of *E. coli* transformants, F199, JMN2, JMN123, and JMN159 were grown in LB for 12 h (*E. coli* strains) and 24 h (F199 and F199-derivative strains) at 30°C with linear shaking at 160 rpm. F199 derivatives were cultured in Wx medium containing 10 mM sucrose, 10 mM glutamate, 0.13 mM methionine, and 10 mM proline (Wx-SEMP) and Wx-SEMP containing 2 mM GGE, 2 mM β-HPV, or 2 mM guaiacol for 12 h at 30°C. The cells were collected by centrifugation (14,000 x *g* for 1 min), washed twice with 50 mM Tris-HCl buffer (pH 7.5), resuspended in the same buffer, and used as resting cells.

### Conversion of β-HPV by F199, F199-derivative strains, and *E*. *coli* expressing *hpvY*

Resting cells of F199, JMN2, JMN123, JMN159, and *E. coli* BL21(DE3) harboring p16hpvY (F199 and F199-derivative strains, OD_600_ of 5.0; *E. coli* transformant, OD_600_ of 10) were incubated with 100 µM HPV at 30°C with shaking for 8–24 h. The supernatant obtained by centrifugation at 19,000 × *g* for 1 min at 4°C was analyzed by HPLC.

### Expression of *hpvY* in *E. coli*

A DNA fragment carrying *hpvY* (RS19130) was amplified through PCR using the F199 total DNA and the primer pairs listed in Table 2. The amplified fragment of *hpvY* was cloned into pET-16b using a NEBuilder HiFi DNA assembly cloning kit to form p16hpvY. *E. coli* BL21(DE3) cells harboring p16hpvY were grown in LB, and gene expression was induced for 4 h at 30°C by adding 1 mM isopropyl-β-D-thiogalactopyranoside when the OD_600_ of the culture reached 0.5. Gene expression was examined using sodium dodecyl sulfate–12% polyacrylamide gel electrophoresis. The protein bands in gels were stained with Coomassie brilliant blue (35).

**TABLE 2:** Primers used in this study

Purpose	Primer name	Sequences (5′–3′)
Plasmid construction		
p16hpvY	p16hpvY_F	TCGAAGGTCGTCAATGGCCGAGGCAGCGGGCGA
	p16hpvY_R	GTTAGCAGCCGGATCCTCAGACGAAGTCCACCGTCC

### HPLC analysis

HPLC analysis was conducted using the ACQUITY UPLC system (Waters). The sample was filtered through a PTFE filter (Captiva Econofilter, Agilent) with a pore size of 0.20 µm and then analyzed using a TSKgel ODS-140HPT column (particle size, 2.3 µm; 2.1 × 100 mm, Tosoh). Analysis of HPV, VAL-Tris, and vanilloyl acetic acid (VAA) was performed in gradient mode. The mobile phase was a mixture of solution A (acetonitrile containing 0.1% formic acid) and B (water containing 0.1% formic acid) under the following conditions: 0–3.2 min, 5% A; 3.2–6.0 min, linear gradient from 5.0% to 40% A; 6.0–6.5 min, decreasing gradient from 40% to 5.0% A; 6.5–7.0 min, 5% A. The flow rate was 0.5 mL/min, and the column temperature was 30°C. HPV, VAL-Tris, and VAA were detected at 310 nm.

### Proteomics analysis

Cultures of F199, JMN2, and JMN123 were grown overnight in DSM 457 medium with 1 g/L glucose. These cultures were then diluted 1:100 into fresh medium in triplicates containing either 1 g/L glucose or 1 g/L GGE. After growth to the mid-log phase (OD_600_ 0.4–0.6), 1.5 mL of cells was collected and pelleted by centrifugation for 1 min at 10,000 × *g*, washed in 1 mL DSM 457 medium without carbon, pelleted again, and flash frozen in liquid nitrogen.

Cells were then prepared for proteomic analysis as previously described (36). Briefly, cells were lysed by bead beating with 0.15 mm zirconium oxide beads in 100 mM Tris-HCl, pH 8.0 (Geno/Grinder 2010; SPEX). The crude lysates were adjusted to 4% SDS and 10 mM dithiothreitol, heated to 90°C, and pre-cleared by centrifugation. Protein concentrations were measured by a Nanodrop OneC spectrophotometer at 205 nm (with 20× dilution with water). Pre-cleared lysates were moved to a new tube, and cysteines alkylated with 30 mM iodoacetamide. Proteins were isolated via the protein aggregation capture method (37), washed as prescribed, and digested *in situ* with sequencing-grade trypsin at a 1:75 (wt/wt) trypsin-to-protein ratio in 100 mM ammonium bicarbonate, pH 8.0, at 37°C overnight and the following day. The resulting tryptic digests were acidified with formic acid, filtered through a 10 kDa MWCO spin filter (Vivaspin500; Sartorius), and quantified by NanoDrop.

Peptide samples were analyzed by automated 1D LC-MS/MS using a Vanquish UHPLC connected directly to a nanoelectrospray source on a Q Exactive Plus mass spectrometer (Thermo Scientific). For each sample, 2 µg of peptides was loaded directly on an in-house pulled nanospray emitter (75 micron ID) packed with 15 cm of Kinetex RP C18 resin (1.7 micron; Phenomenex) and separated by UHPLC along a 180-min organic gradient (36). Eluting peptides were measured and sequenced by data-dependent acquisition on the MS (38). MS/MS spectra were searched against the *N. aromaticivorans* reference proteome from UniProt (along with common protein contaminants) using the SEQUEST HT algorithm in Proteome Discoverer version 2.5 (Thermo Scientific), and peptide spectrum matches (PSMs) were scored and filtered with Percolator, controlling the false-discovery rate to <1% at the PSM and peptide levels (39). Peptides were quantified by the chromatographic area under the curve, mapped to their respective proteins, summed to estimate protein-level abundance, and statistically processed as previously described (39).

## RESULTS AND DISCUSSION

### Laboratory evolution of F199 for growth with lignin-derived aromatics

We previously described the evolution and resequencing of strain JMN2, a mutant of F199 that was selected for growth with GGE as the sole carbon source (17, 21). To identify other potential mutations that improve growth with GGE, we initiated additional evolution experiments. Three independent cultures of F199 were grown and serially passaged in a minimal medium containing GGE as the sole carbon source. Spontaneous mutations are expected to occur at random during growth, and those that provide a benefit during growth with GGE will become enriched in the population. After five transfers, each mixed evolution culture showed an improved growth rate and yield compared to the F199 parent. We then isolated approximately eight single colonies from each replicate culture and assayed growth with GGE as the sole carbon and energy source. Each culture yielded at least one isolate that showed a higher final optical density when grown with GGE. We selected one strain from each replicate culture, described hereafter as JMN121, JMN122, and JMN123, for further analysis (Fig. 2A; Fig. S1). One evolved strain, JMN123, had higher final optical densities than JMN2 when grown with GGE but not with a related lignin-derived aromatic compound, ferulate (Fig. 2B).

### Identification of mutations in evolved lineages

To gain insight into the genetic basis for the evolved phenotypes, we resequenced the genomes of JMN121, JMN122, and JMN123 using short reads and, to detect any large-scale rearrangements, generated a *de novo* genome sequence of JMN123 using Nanopore sequencing. No large rearrangements were identified, and a full comparison of the various point mutations found in these strains is shown in Table S1. All four strains contain parallel mutations, including large deletions in plasmid pNL1. The growth profiles with GGE were similar between JMN2, JMN121, and JMN122, so only JMN2 and JMN123 were analyzed in later experiments.

### Role of *ligE* in GGE metabolism

Upon examination and comparison of the mutations found within the four evolved strains, we noted that three of the four strains contain an identical point mutation 55 bp upstream of the start codon of *Saro_RS12100*. This gene encodes LigE, one of the β-etherases known to be involved in GGE catabolism (10, 13). Based on sequence analysis, this upstream region contains plausible −35 and −10 promoter sites as well as an inverted repeat motif (Fig. S2). The observed point mutation occurs in the predicted inverted repeat motif and could potentially interfere with the binding of a transcriptional regulator (40). Repeated attempts to reconstruct this single nucleotide mutation in a wild-type background were unsuccessful. Instead, an in-frame unmarked deletion of *ligE* was engineered in both JMN2 and JMN123, and the resulting strains were assessed for their growth in minimal medium with GGE. In both genetic backgrounds, the *∆ligE* mutation severely hindered growth with GGE (Fig. S3). To better understand the effects of the *ligE* mutation, we performed a global proteomics analysis of F199, JMN2, and JMN123 grown with glucose or GGE. Since F199 grew very slowly with GGE (Fig. 2A), we only analyzed F199 grown with glucose. In both JMN2 and JMN123, LigE expression during growth with glucose was approximately 1,000-fold higher than in F199 grown with glucose, and LigE expression increased further during growth with GGE (Fig. 3A). We propose that the *ligE* mutation was most likely a gain of function mutation that increases basal LigE expression and thereby improves GGE conversion to monomers.

### Effects of deletions in pNL1

Wild-type F199 contains a 184 kb plasmid, pNL1, with many accessory catabolic genes (41). Based on previous sequencing and genetic characterization of pNL1, this plasmid can be divided into three distinct regions based on predicted gene functions within each region: replication, mobilization, and aromatic degradation. All four evolved strains contained significant deletions in the aromatic degradation region, which contains a catechol-2,3 cleavage pathway (*xylEGHIJKQ*) (Fig. S4). This *xyl* pathway was shown to contribute to the degradation of protocatechuate in a previous study focused on aromatic pathway discovery in F199 (22).

Slightly more of the aromatic degradation region was deleted in JMN2 than in JMN123. We hypothesized that this genetic difference could explain the improved yield of JMN123 during growth with GGE. To test this hypothesis, we made an additional targeted deletion in pNL1 of JMN123 to replicate the deletion seen in JMN2 (Fig. S5A), yielding strain JMN151. Compared to JMN123, JMN151 reached a slightly lower optical density and had a slightly longer lag phase during growth with GGE but was still substantially improved compared to JMN2 (Fig. S5B). We conclude that differences in the extent of deletion of pNL1 may affect growth with GGE but are not the major contributor to the improved growth by strain JMN123.

### Identification of a new enzyme and characterization of its role in GGE catabolism

Further comparison of the mutations found in the four evolved isolates identified a single nucleotide mutation that was found in JMN121 and JMN123, 49 bp upstream of the start codon of *Saro_RS19130*. As with the *ligE* mutation, this mutation is found in a region upstream of the coding sequence of *RS19130* that contains a predicted promoter (Fig. S6). This gene encodes a predicted choline dehydrogenase that has 39% amino acid sequence identity to HpvZ from SYK-6. Based on this homology, we refer to *RS19130* as *hpvY*. We again used global proteomics to understand the effect of this mutation on HpvY expression (Fig. 3B). During growth with glucose, expression was similar for F199 and JMN2 but increased 2,400 ± 200-fold in JMN123 compared to F199. During growth with GGE, HpvY expression was induced 32 ± 2-fold in JMN2 and a further 2-fold in JMN123.

**Fig 4:**
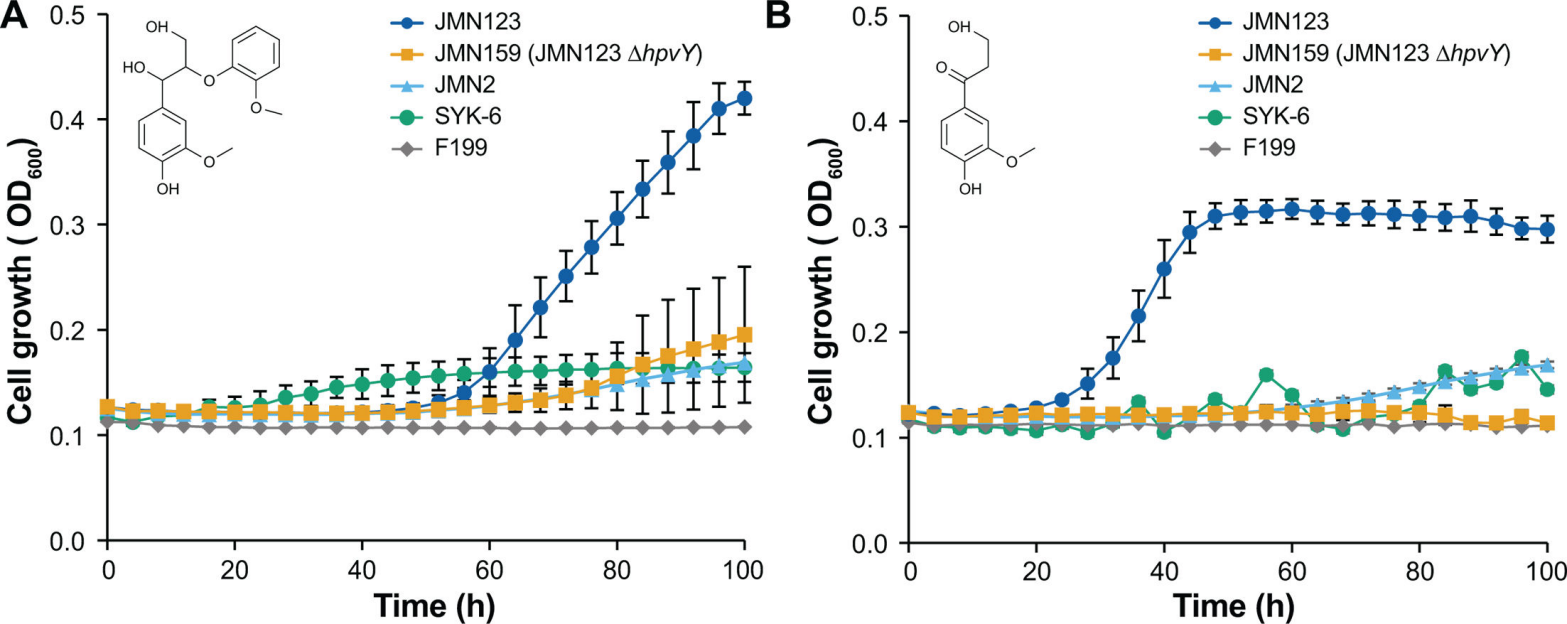
Impact of *hpvY* (*RS19130*) deletion during growth with GGE and β-HPV. (**A**) Deletion of *hpvY* from JMN123, yielding JMN159, significantly decreases growth yield with GGE. Strains were grown in minimal medium with 2 mM GGE as the sole carbon source. (**B**) Deletion of *hpvY* completely abolishes growth with β-HPV in JMN159. Strains were grown in minimal medium with 2 mM β-HPV as the sole carbon source. In both panels, error bars show the standard deviation calculated from three biological replicates.

To better understand the impact of increased HpvY expression, we constructed an in-frame deletion of *hpvY* in JMN123 to yield strain JMN159. When strains JMN2, JMN123, and JMN159 were grown with GGE as a sole carbon source, JMN159 showed a significant decrease in yield, comparable to JMN2 but still higher than the wild-type F199 (Fig. 4A).

To further identify the role of HpvY in GGE catabolism, we measured the growth of the evolved and engineered strains with β-HPV as the sole carbon and energy source (Fig. 4B). JMN123 grew rapidly with β-HPV, while deletion of *hpvY* in JMN159 completely abolished growth with β-HPV. The growth defect in JMN159 was observed with GGE and β-HPV but not with a related lignin-derived aromatic compound, ferulate (Fig. S7). We conclude that the mutation in JMN123 upstream of *hpvY* activated a previously unidentified enzyme for β-HPV catabolism. Since deletion of *hpvY* in JMN159 prevents growth with β-HPV, we hypothesize that residual growth of JMN159 with GGE is due to catabolism of GGE-derived guaiacol, similar to JMN2.

**Fig 5:**
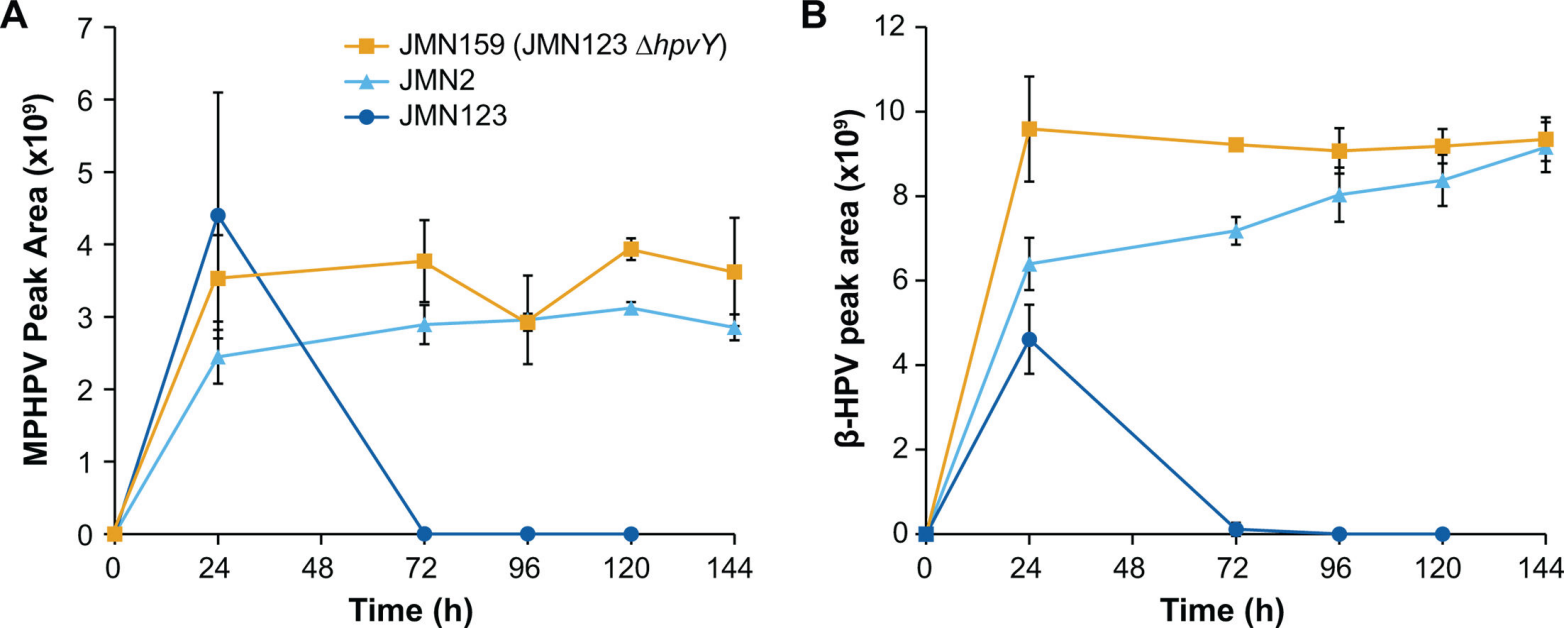
Impact of *hpvY* (*RS19130*) deletion on GGE conversion. Cells were grown in minimal medium containing 1 g/L GGE (3.1 mM) as the sole carbon source. Accumulation of (**A**) MPHPV and (**B**) β-HPV was monitored by LC-MS. Error bars show one standard deviation, calculated from three biological replicates.

We also monitored metabolite accumulation during growth with GGE to identify pathway bottlenecks that were introduced or removed by alterations to *hpvY*. Similar to the previous experiments, we grew JMN2, JMN123, and JMN159 with GGE as the sole source of carbon and energy and monitored the concentration of intermediate metabolites MPHPV and β-HPV (Fig. 5). JMN2 accumulated significant concentrations of both intermediates, while JMN123 accumulated these intermediates only transiently and then fully consumed them. Deletion of *hpvY* in JMN159 again led to the accumulation of these intermediates, consistent with a role for HpvY in β-HPV catabolism. Since HpvY is likely involved in β-HPV catabolism, but deletion of *hpvY* leads to the accumulation of the upstream metabolite MPHPV rather than β-HPV, we hypothesize that accumulated β-HPV inhibited the glutathione transferases responsible for the conversion of MPHPV to β-HPV, resulting in MPHPV accumulation.

### Role of HpvY in β-HPV catabolism

**Fig 6:**
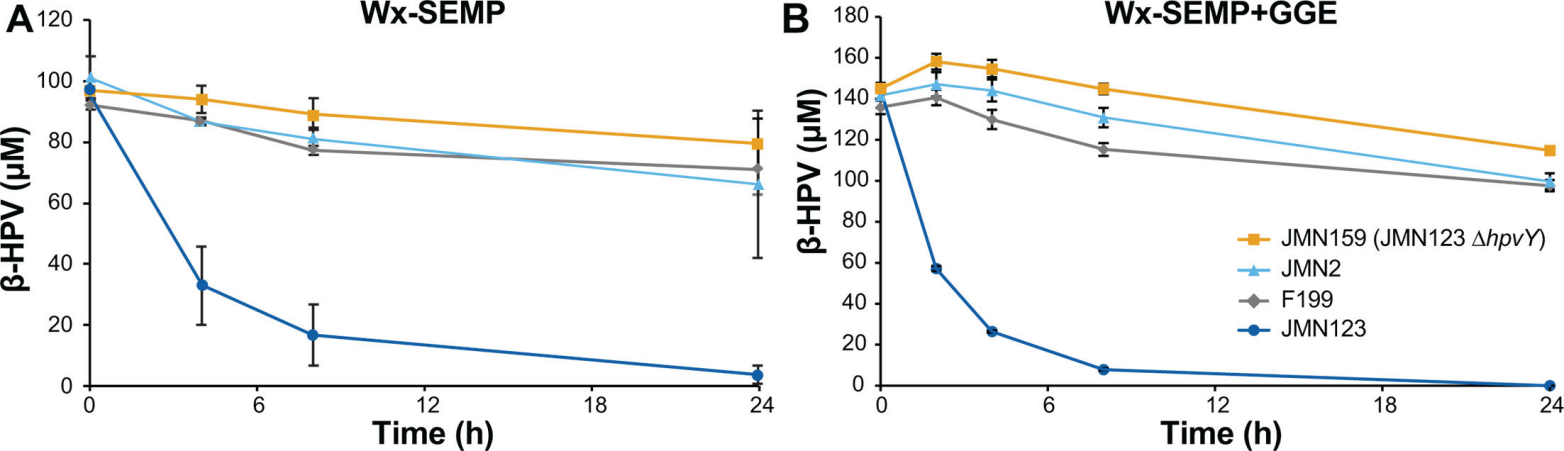
Conversion of β-HPV by resting cells of *N. aromaticivorans*. Strains were pre-cultured in (**A**) Wx-SEMP or (**B**) Wx-SEMP + 2 mM GGE, concentrated, and incubated with β-HPV. Residual β-HPV was detected by HPLC. Error bars show the standard deviation, calculated from three biological replicates.

Low accumulation of β-HPV during growth with GGE suggested that JMN123 has higher β-HPV conversion activity than the other *N. aromaticivorans* strains tested. To more directly measure β-HPV conversion and test the regulation of this activity, we grew strains of *N. aromaticivorans* with four different media, harvested the cells, added β-HPV, and measured the disappearance of this substrate by HPLC. When cells were grown in Wx-SEMP medium with or without 2 mM GGE, β-HPV conversion was observed by JMN123 but not by JMN159 (JMN123 Δ*hpvY*) (Fig. 6). We conclude that *hpvY* is required for β-HPV conversion and that this activity is constitutive in JMN123.

### Heterologous expression of HpvY and demonstration of β-HPV conversion

**Fig 7:**
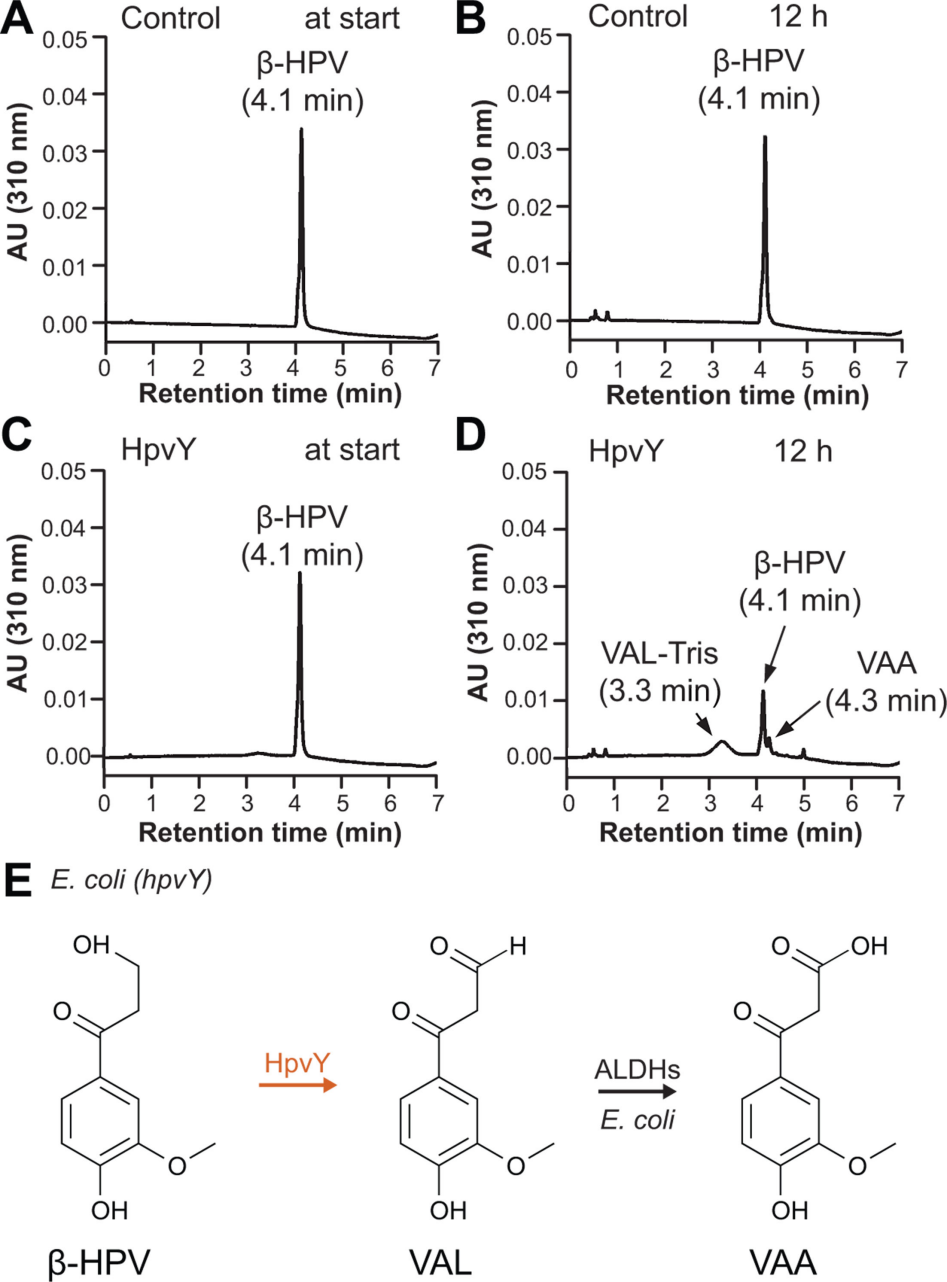
HPLC analysis of β-HPV conversion by resting *E. coli* cell suspensions containing either an empty plasmid control (A and B) or a plasmid heterologously expressing *hpvY* (C and D). 100 µM β-HPV was added at *t*_0_. β-HPV, VAA, and VAL-Tris are identified by comparison to authentic standards. (**E**) Proposed reaction scheme in *E. coli* heterologously expressing HpvY. β-HPV, β-hydroxypropiovanillone; VAL, vanilloyl acetaldehyde; VAL-Tris, imine derivative of VAL produced non-enzymatically by reaction with Tris (20); VAA, vanilloyl acetic acid.

While β-HPV conversion by JMN123 requires *hpvY*, the effect of HpvY on β-HPV conversion could be indirect. To identify the activity of HpvY, we heterologously expressed *hpvY* in *Escherichia coli*. We did not attempt to purify HpvY, as our previous attempts to purify active HpvZ from *E. coli* were unsuccessful, likely due to its membrane association (20). We observed the expression of a protein with the expected mass of approximately 60 kDa in both the soluble and insoluble fractions (Fig. S8). We, therefore, measured the conversion of β-HPV using resting cells of control and *hpvY*-expressing *E. coli*. No conversion of β-HPV was observed by the control cells, while the cells expressing HpvY produced VAL and VAA (Fig. 7). VAA is likely produced through promiscuous oxidation of VAL by endogenous aldehyde dehydrogenases (Fig. 1). Based on these results, we conclude that HpvY directly oxidizes β-HPV to VAL, equivalent to the reaction catalyzed by HpvZ (20). A precise comparison of catalytic properties between HpvY and HpvZ would require additional enzyme purification.

Similar to SYK-6 and resting *E. coli*, it is likely that VAL produced by HpvY in F199 is oxidized to VAA. In SYK-6, VAA is then converted to VA by the actions of VceA, VceB, and an unidentified enzyme. However, homologs of VceA and VceB are not found in F199, suggesting that additional pathway enzymes remain to be discovered.

### Conclusion

In this work, we used adaptive laboratory evolution to generate a mutant strain of *N. aromaticivorans* F199 that rapidly and completely catabolizes a model lignin dimer. Resequencing multiple evolved strains identified several key parallel mutations that contribute to the improved growth phenotype. Further analysis of these mutations led to the discovery of an uncharacterized β-HPV-converting enzyme, designated HpvY, that converts β-HPV into VAL. Continued pathway discovery efforts are needed to identify the remaining enzymes in F199 for VAL catabolism. Additionally, we have shown that the growth of F199 with GGE can be improved through experimental evolution, but it is not yet clear whether the same is true of SYK-6. Further work is needed to identify whether differences between JMN123 and SYK-6 during growth with GGE are due to differences in enzyme catalytic efficiency, for example, between HpvZ and HpvY, or whether SYK-6 could grow equally well with GGE after a similar degree of evolutionary optimization. This work highlights the utility of experimental evolution for both pathway optimization and pathway discovery.

## Data Availability

Sequencing reads for strains JMN121, JMN122, and JMN123 are available from the Sequence Read Archive through BioProject PRJNA1108622. Proteomics data are available at MassIVE (accession MSV000095729).

## SUPPLEMENTAL MATERIAL

Supplemental figures aem.02081-24-s0001.pdf Figures S1 to S8.

Table S1 aem.02081-24-s0002.xlsx Mutations identified in evolved strains.

ASM does not own the copyrights to Supplemental Material that may be linked to, or accessed through, an article. The authors have granted ASM a non-exclusive, world-wide license to publish the Supplemental Material files. Please contact the corresponding author directly for reuse.
