# Supplementary material for: Laboratory evolution in Novosphingobium aromaticivorans enables rapid catabolism of a model lignin-derived aromatic dimer
Source: Appl Environ Microbiol. 2025 Jan 23;91(2):e02081-24. doi: 10.1128/aem.02081-24 (PMC11837543; doi:10.1128/aem.02081-24)
Supplement: Supplemental figures — Figures S1 to S8. [file aem.02081-24-s0001.pdf]

Supplemental Information

Laboratory evolution in *Novosphingobium aromaticivorans* enables rapid catabolism of a model lignin-derived aromatic dimer

Marco N. Allemann[1,‡], Ryo Kato[2], Dana L. Carper[1,3], Leah H. Hochanadel[1], William G. Alexander[1], Richard J. Giannone[1,3], Naofumi Kamimura[2], Eiji Masai[2], and Joshua K. Michener[1,†]

[1]Biosciences Division, Oak Ridge National Laboratory, 1 Bethel Valley Rd, Oak Ridge, TN 37830, USA. [2]Department of Materials Science and Bioengineering, Nagaoka University of Technology, Nagaoka, Niigata, 940-2188, Japan. [3]Center for Bioenergy Innovation, Oak Ridge National Laboratory, Oak Ridge, TN, 37830.

[‡]Present address: Quorum Bio, New York, New York.

[†]To whom correspondence should be addressed: michenerjk@ornl.gov

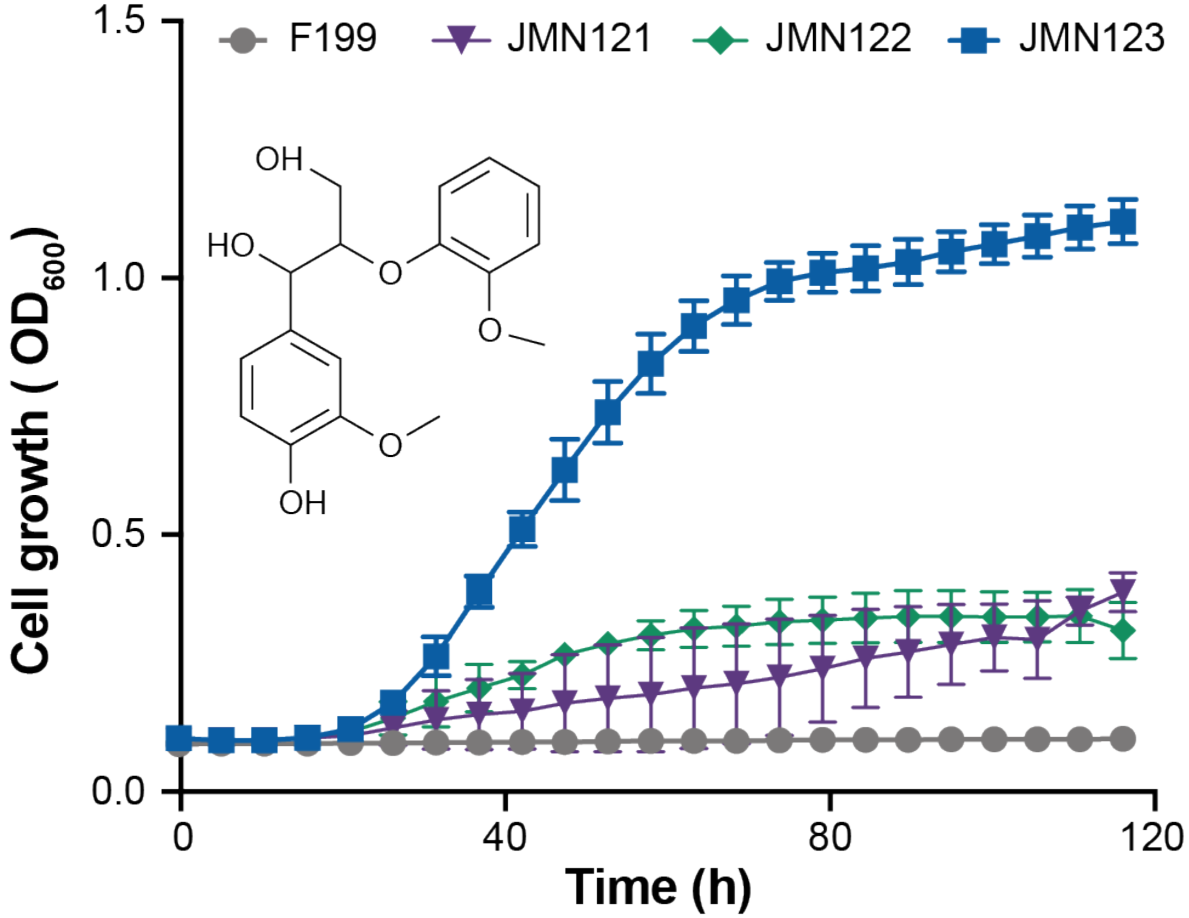

Figure S1: Growth of wild-type and evolved strains with 1 g/L GGE (3.1 mM) as the sole carbon and energy source. Error bars show one standard deviation, calculated from three independent experiments.

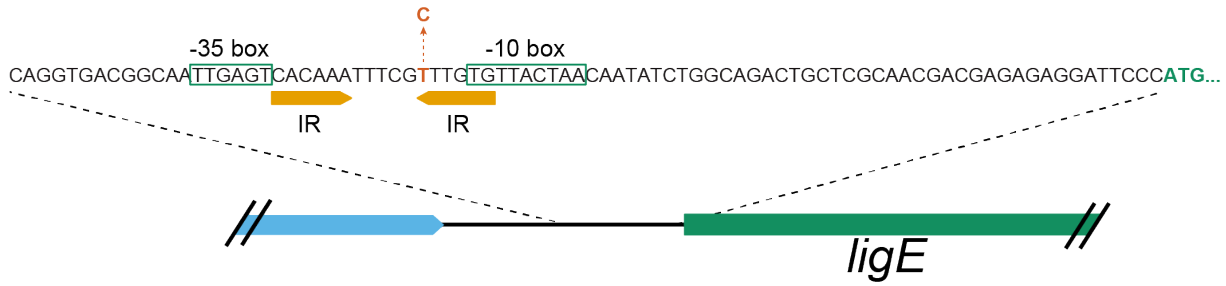

Figure S2: Parallel mutations upstream of *ligE* disrupt an inverted repeat in the predicted promoter. The chromosomal organization and predicted promoter region is shown for *ligE*. The indicated T→C mutation was identified in all four evolved variants and disrupts an inverted repeat.

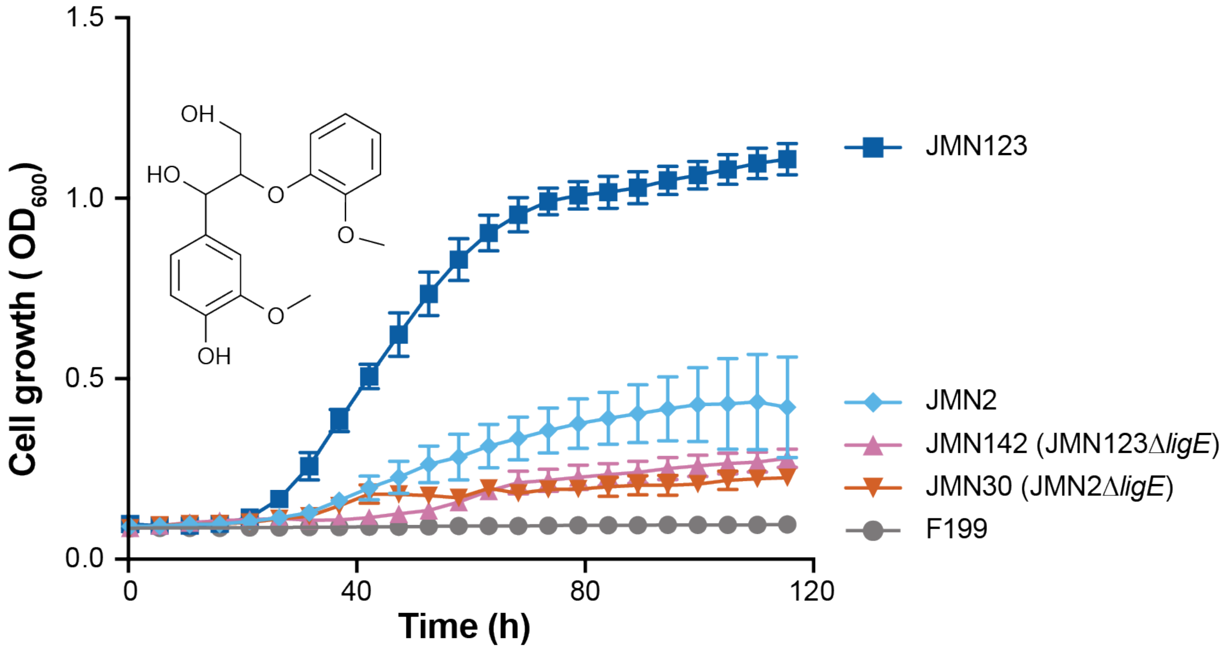

Figure S3: Growth of wild-type (F199), evolved (JMN2, JMN123), and engineered (JMN30, JMN142) strains with 1 g/L GGE (3.1 mM) as the sole carbon and energy source. Error bars show one standard deviation, calculated from three independent experiments.

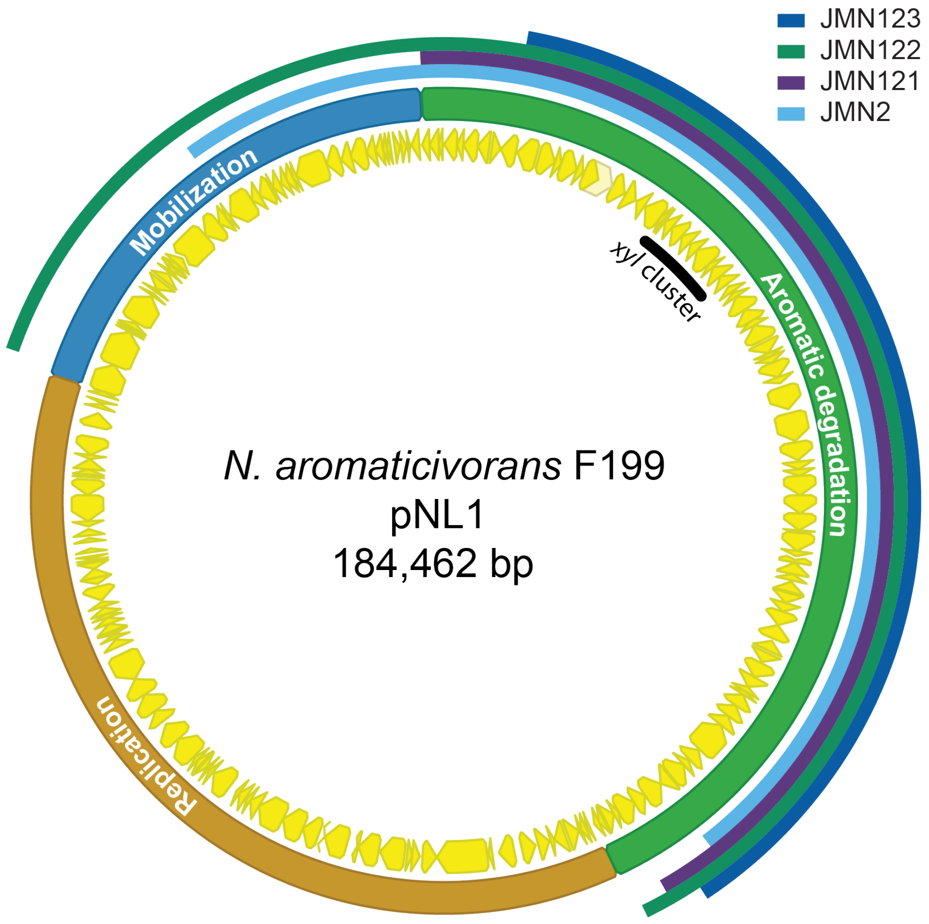

Figure S4: Parallel deletions to accessory plasmid pNL1 were observed in all four evolved isolates, though the extent of deletion varied between strains. Outside colored circles show the region deleted in each evolved strain. The *xyl* catabolic locus is highlighted inside the circle. The plasmid replication region was maintained in all four strains.

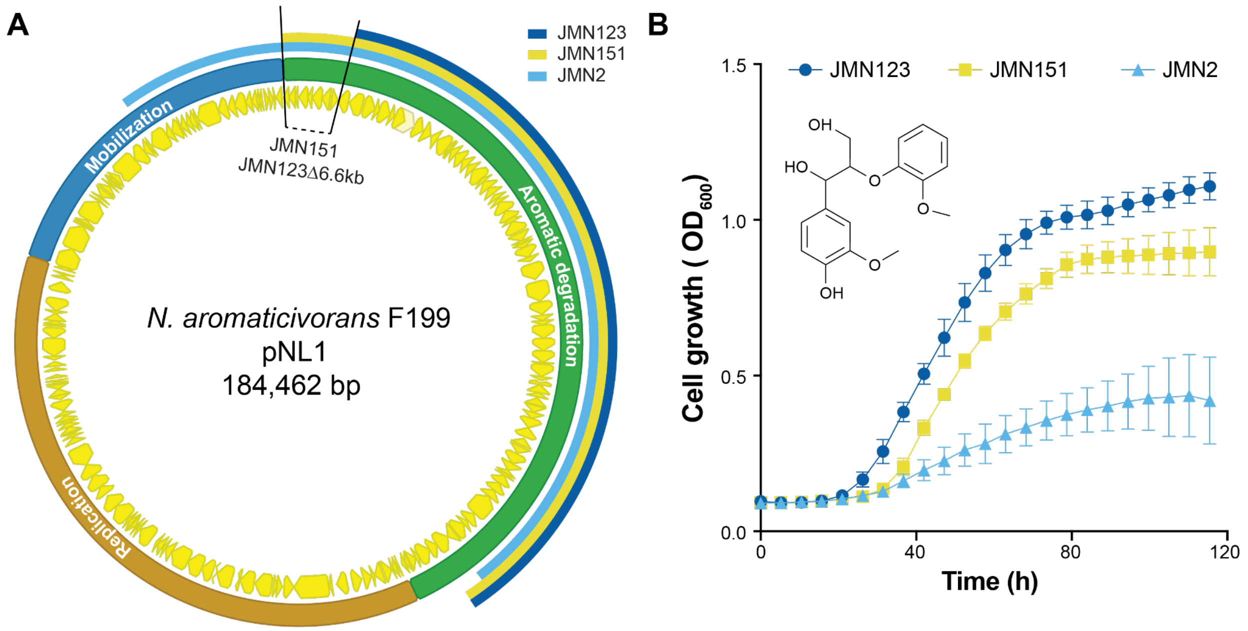

Figure S5: (A) An additional portion of the aromatic degradation region of pNL1 was deleted from JMN123, yielding strain JMN151. (B) Growth of evolved (JMN2 and JMN123) and mutant (JMN151) strains with 1 g/L GGE as the sole source of carbon and energy. Error bars show one standard deviation, calculated from three independent experiments.

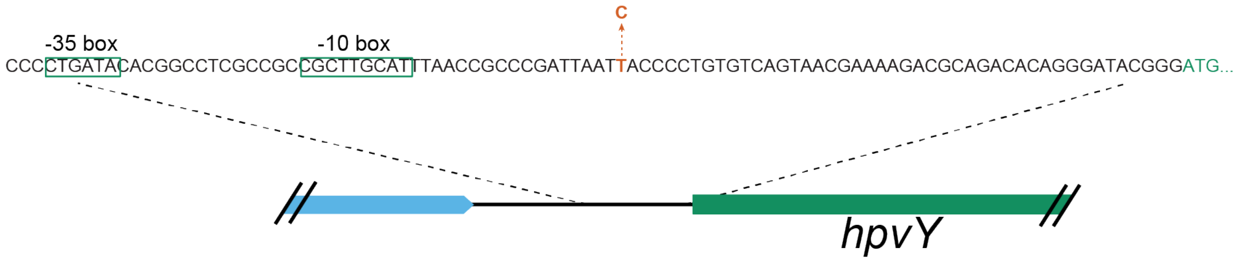

Figure S6: JMN123 contains a single nucleotide mutation 5’ of the *hpvY* coding region, between the putative promoter and start codon.

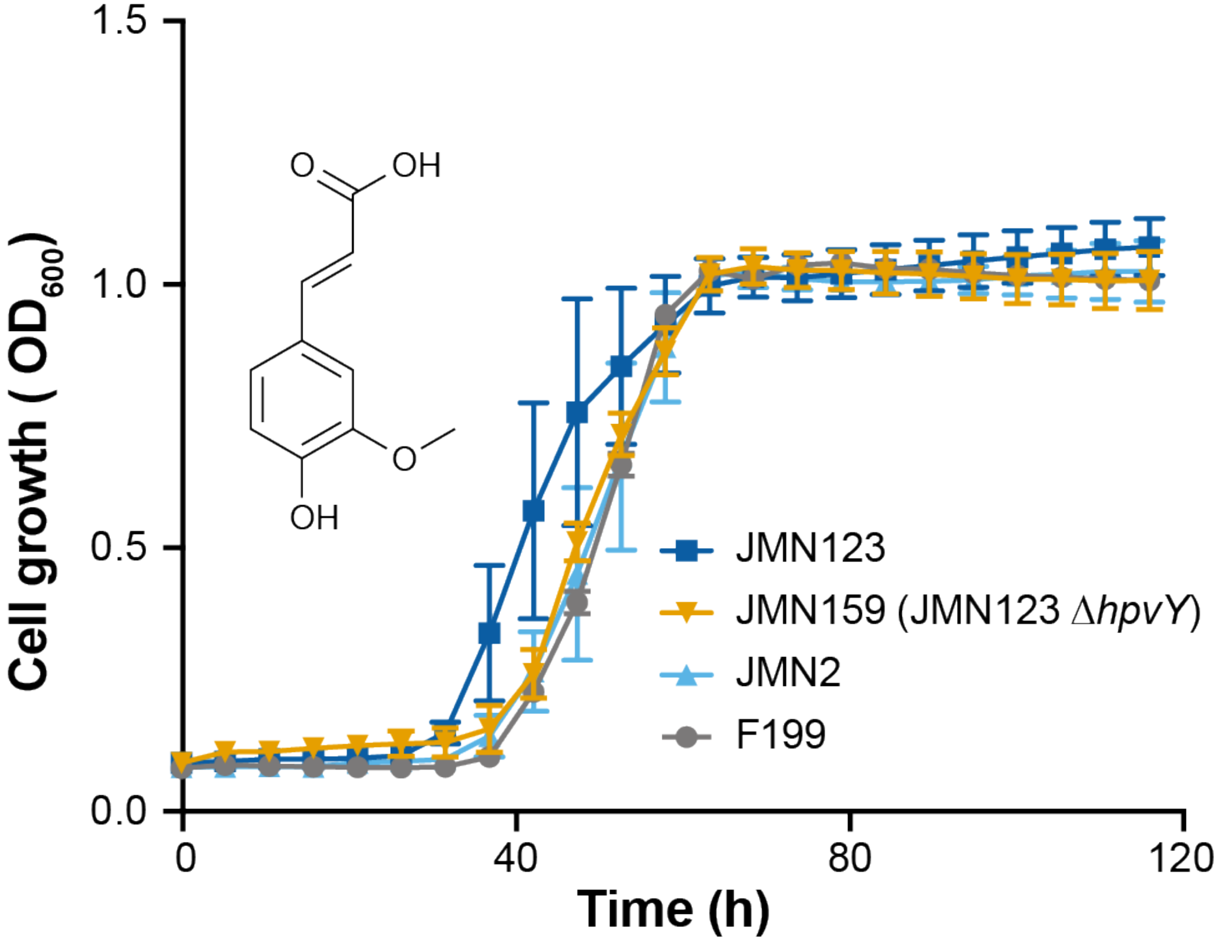

Figure S7: Growth of wild-type (F199), evolved (JMN2, JMN123), and mutant (JMN159) strains with 1 g/L ferulate (5.1 mM) as the sole source of carbon and energy. Error bars show one standard deviation, calculated from three independent experiments.

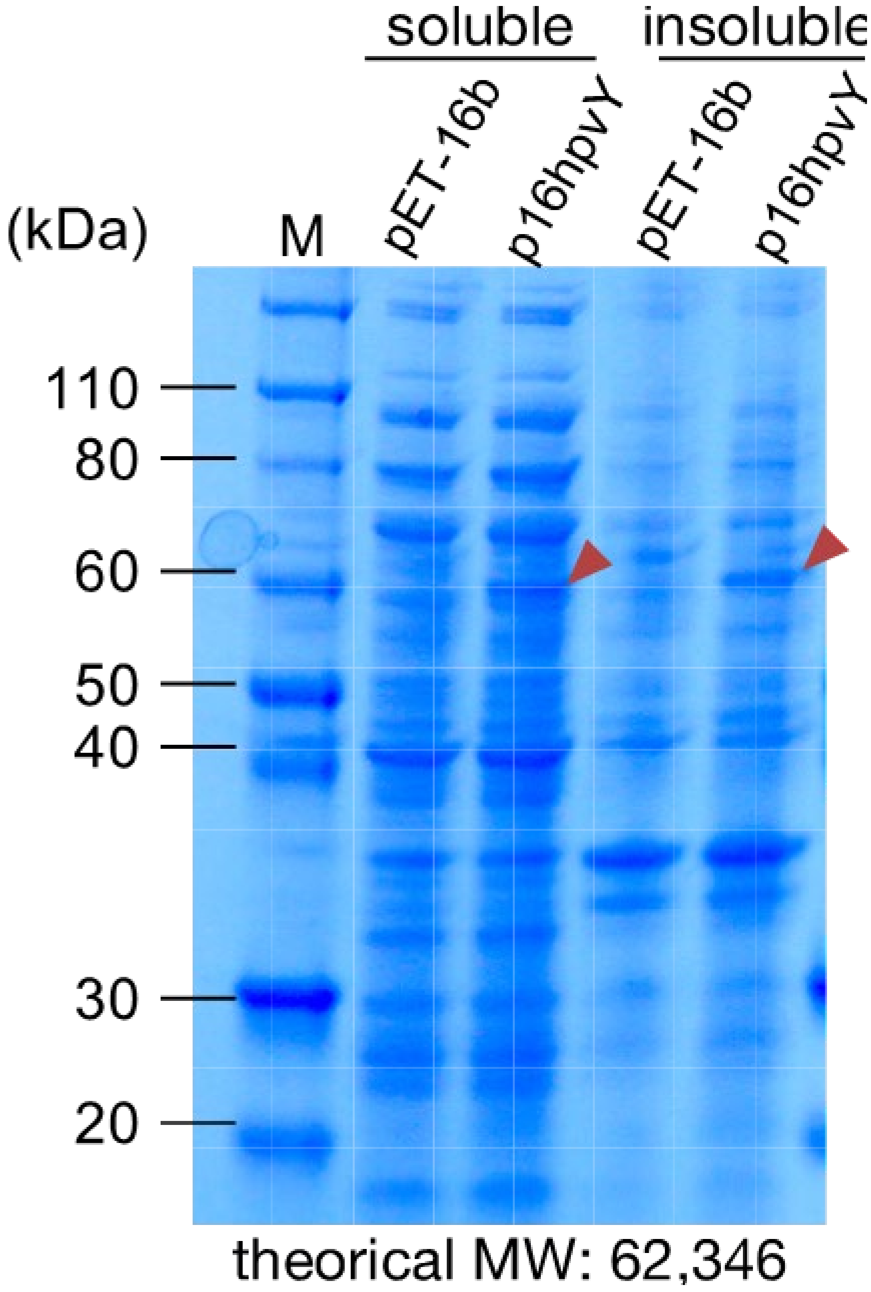

Figure S8: Heterologous expression of *hpvY* in *E. coli*. The associated gene was expressed from pET-16b with an N-terminal His tag. A protein of the expected mass was observed in both the soluble and insoluble fractions of the associated cell lysate.
